# Development and analysis of a 20K SNP array for potato (*Solanum tuberosum*): an insight into the breeding history

**DOI:** 10.1007/s00122-015-2593-y

**Published:** 2015-08-12

**Authors:** Peter G. Vos, Jan G. A. M. L. Uitdewilligen, Roeland E. Voorrips, Richard G. F. Visser, Herman J. van Eck

**Affiliations:** Wageningen UR, Plant Breeding, P.O. Box 386, 6700 AJ Wageningen, The Netherlands; Centre for BioSystems Genomics, P.O. Box 98, 6700 AB Wageningen, The Netherlands

## Abstract

*****Key message***:**

**A 20K SNP array was developed and a comprehensive set of tetraploid cultivar was genotyped. This allowed us to identify footprints of the breeding history in contemporary breeding material such as identification of introgression segments, selection and founder signatures.**

***Abstract*:**

A non-redundant subset of 15,138 previously identified SNPs and 4454 SNPs originating from the SolCAP project were combined into a 20k Infinium SNP array for genotyping a total of 569 potato genotypes. In this study we describe how this SNP array (encoded SolSTW array) was designed and analysed with fitTetra, software designed for autotetraploids. Genotypes from different countries and market segments, complemented with historic cultivars and important progenitors, were genotyped. This comprehensive set of genotypes combined with the deliberate inclusion of a large proportion of SNPs with a low minor allele frequency allowed us to distinguish genetic variation contributed by introgression breeding. This “new” (post 1945) genetic variation is located on specific chromosomal regions and enables the identification of SNP markers linked to R-genes. In addition, when the genetic composition of modern cultivars was compared with cultivars released before 1945, it appears that 96 % of the genetic variants present in those ancestral cultivars remains polymorphic in modern cultivars. Hence, genetic erosion is almost absent in potato. Finally, we studied population genetic processes shaping the genetic composition of the modern European potato including drift, selection and founder effects. This resulted in the identification of major founders contributing to contemporary germplasm.

**Electronic supplementary material:**

The online version of this article (doi:10.1007/s00122-015-2593-y) contains supplementary material, which is available to authorized users.

## Introduction

The genetic diversity of cultivated potato, as studied today, is easier to interpret with insights from the past events that have shaped the gene pool. These past events include (1) the amount of genetic variation that was brought from South America to Europe since the late 16th century (Hawkes and Francisco-Ortega 1993), (2) the loss of diversity during the late blight epidemics in the 19th century, and more recently (3) introductions of (wild) Latin American species contributing to pathogen resistance, and (4) the more focussed breeding for specific niche markets. These genetic interventions leave their traces and can be recognized using modern DNA tools. Moreover, the maintenance of the original named cultivars via clonal reproduction allows the historical analysis of the breeding process by comparing their genetic makeup with contemporary cultivars.

A number of historic cultivars dating back to the 19th century are still widely grown, as progress in cultivar improvement is limited due to the low reproduction rate and complex autotetraploid inheritance (2*n* = 4*x* = 48). Examples of historic cultivars are Russet Burbank (1908), a Burbank (1876) mutant, the most important cultivar in the USA and Bintje (1910) ranking 1st in Belgium and 6th in the Netherlands in 2014. Throughout the 100 years of breeding hardly any increase in yield has been achieved (Douches et al. 1996), nevertheless major improvements have been made by introgression of resistance genes. Introgression breeding, practiced from the early 20th century onwards, focussed on late blight, cyst nematodes and viruses using *S. demissum*, *S. stoloniferum*, *S. tuberosum* Group Andigena clone CPC-1673 and *S. vernei* reviewed by Bradshaw and Ramsay (2005). This review describes the utilization of the Commonwealth Potato Collection (CPC) material, and similar work was performed in Germany and the Netherlands using other germplasm collections, e.g. Braunschweig Genetic Resource Collection (BGRC). Markers have been developed for the vast majority of the important resistance genes. Recent genepool-wide validation studies that aim to predict the presence of multiple resistances are still being performed with single locus marker techniques (Lopez-Pardo et al. 2013; Sharma et al. 2014). Unfortunately, it will take an additional effort to convert gel-based markers into SNP assays suitable for highly parallel genotyping methods.

In contrast to breeding for resistance, marker-assisted breeding for yield and tuber quality traits is still in its infancy. Marker-assisted breeding for such traits with a quantitative and polygenic nature will require a much deeper understanding of the loci and the beneficial and deleterious alleles involved. Genomic selection however does not rely on such information, but for both strategies the implementation of SNP arrays is urgent, to allow sufficient data collection to improve breeding efficiency. One of the first examples of highly parallel marker studies in potato made use of methods such as the Golden Gate assay (Anithakumari et al. 2010), the KASP SNP genotyping system (http://www.kbioscience.co.uk) (Lindhout et al. 2011) and more recently a SNP array with 8303 markers was developed (Felcher et al. 2012; Hamilton et al. 2011), which is currently widely used in potato research (Hirsch et al. 2013; Manrique-Carpintero et al. 2014; Prashar et al. 2014).

Such a SNP array requires a SNP discovery study, which is facilitated by next-generation sequencing. Different approaches for SNP discovery are used such as (1) whole genome resequencing (Yamamoto et al. 2010), (2) transcriptome sequencing (Barbazuk et al. 2007; Hamilton et al. 2011, 2012; Trick et al. 2009) and (3) reduced representation sequencing based on restriction enzymes (Baird et al. 2008). These studies do not need any prior knowledge of a reference genome. The study of Uitdewilligen et al. (2013) used the potato reference genome (PGSC 2011) to perform a targeted resequencing of a subset of 800 genes (2.1 Mb) from the potato genome.

Many of these SNP discovery studies are based on a few genotypes, for example the parents of an important mapping population (Bundock et al. 2009), one rice cultivar compared to the reference genome (Yamamoto et al. 2010), six potato cultivars (Hamilton et al. 2011) or four tomato cultivars combined with two wild relatives (Sim et al. 2012). Even the most commonly used array in *Arabidopsis* was based on the sequence of only 19 accessions (Kim et al. 2007). This could result in an ascertainment bias when this array is applied on a much wider or different germplasm (Moragues et al. 2010; Thomson et al. 2012). In more recent studies larger SNP discovery panels are sequenced, for example in wheat (Wang et al. 2014). Also in potato a relatively large panel representative for the worldwide gene pool has been used for targeted resequencing (Uitdewilligen et al. 2013). These discovery studies on a wider gene pool are more suitable for the development of large arrays and can be applied to a much wider germplasm.

In this paper we describe the design of a potato 20K SNP array using the two major discovery studies available in potato (Uitdewilligen et al. 2013; Hamilton et al. 2011). On this SNP array a large number of relatively rare variants from Uitdewilligen et al. (2013) have been included. The array was used to genotype a comprehensive panel of 569 genotypes, which included many historically important cultivars and progenitors, from different origin and market niches. We describe the analysis of this array with fitTetra (Voorrips et al. 2011) and subsequently we explore the (changes in the) genetic composition of the potato genepool. This allowed us to (1) identify introgression segments of different origin, (2) study the impact of breeding on allele frequencies in modern germplasm.

## Materials and methods

### Development of SolSTW array

A 20K SNP array was developed predominantly using a subset of the DNA sequence variants as described by Uitdewilligen et al. (2013). Several design criteria were taken into consideration to minimize the risk of assay failure due to flanking polymorphisms, and to maximize the ability to capture haplotypes across the diversity of potato germplasm.

According to the manufacturer’s instructions an Illumina SNP assay has to be free from flanking SNP over a region of preferably 60 bp at one side of the SNP to develop an optimal array. The SNPs selected from Uitdewilligen et al. (2013) were chosen to the following criteria (1) no InDels, tri- or quad- SNPs, (2) no InDels in flanking sequence, (3) only SNPs genotyped with a read depth ≥15× in at least 25 cultivars, (4) minimum flanking SNPs free distance is five nucleotide positions, (5) maximum flanking SNPs at position 6–10 = 1, (6) maximum flanking SNPs at position 6–50 = 5 and (7) no Infinium type I assays. If both the left and right flanking sequences passed these criteria, then the side was chosen with the lowest number of SNPs in the first 10, or 25 or 50 bp.

Next to criteria on technical suitability of SNPs, we applied genetic criteria to reduce redundancy of SNPs whilst maximizing the inclusion of SNPs from different haplotypes. To this end the genotyping calls across 83 cultivars from Uitdewilligen et al. (2013) that were used to cluster SNPs with a Kendall tau test and correlated SNPs (*r*^2^ > 0.5) were considered as one cluster. As a next step the clusters were ordered per gene and subsequently two SNPs per cluster per gene were selected. For the clusters without two SNPs within a gene, one SNP per cluster was selected. Finally singletons were added which were genotyped in at least 67 cultivars, had a maximum of 2 flanking SNPs within 25 bp and no flanking SNPs within 10 bp. In this way SNPs are selected over the full length of large introgression segments and we tried to achieve a uniform distribution of SNPs across the length of the genome and across the depth of haplotypes. We did not filter SNPs according to allele frequency as calculated by Uitdewilligen et al. (2013), because our designed SNP array should allow to monitor the potential increase and decrease of both abundant as well as rare alleles during specific breeding efforts for specific market niches. We did not exclude cultivar-unique SNPs, with the exception of the excessive number of 2,688 unique variants observed in cultivar Vitelotte Noire alone. This resulted in the selection of 15,123 SNPs from Uitdewilligen et al. (2013). Furthermore, 37 chloroplast markers were included (supplementary file 1).

**Fig. 1:**
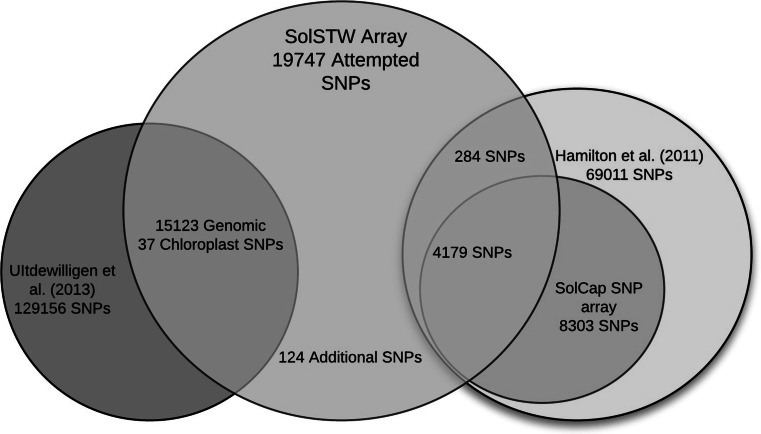
SNP Resources. Venn diagram of SNPs used for development of the SolSTW array (*Orange*). SNPs originated from the 8303 SolCAP array are indicated in *purple*, SNPs originated from Uitdewilligen et al. (2013) are indicated in *blue* and SNP from Hamilton et al. (2011) are indicated in *green*. This figure does not display 87 SNPs included in the SolSTW array, which have been described by both the SNP discovery studies (Uitdewilligen et al. 2013 and Hamilton et al. 2011) (color figure online)

Additionally, we included a subset of 4179 SNPs from the 8303 SolCAP array (Hamilton et al. 2011), which were reported to us to perform well on European tetraploid germplasm (Data not shown). To further improve genome coverage we analysed which PGSC superscaffolds of the potato genome were not yet or insufficiently represented. This resulted in the selection of an additional 284 markers from the 69,011 SolCAP SNPs discovered in Hamilton et al. (2011). Finally, we manually developed 124 SNPs in functional genes involved in morphological and disease resistance traits. In Fig. 1 a Venn diagram is shown of the number of SNPs in each class mentioned above. The figure does not include 87 SNPs which have been found by both the SolCAP and our SNP discovery studies. This Venn diagram shows the attempted numbers of SNPs, but unfortunately, the total number of delivered SNPs was lower as shown in Table 1. To avoid any confusion this 20K SNP array is called the SolSTW array hereafter. It should be noted that Fig. 1 shows the attempted number of SNPs for the SolSTW array and the actual delivered number of SNPs for the SolCAP 8303 array. In supplementary file 1 additional information is specified for all markers as well as assay sequences.

**Table 1:** Numbers of attempted, delivered and successful SNP assays for the SolSTW array separated per SNP discovery study

Origin SNP	Attempted	Delivered	Successful	% Successful
PotVar SNPs (Uitdewilligen et al. 2013)	15,123	13,811	10,707	77.5
Chloroplast SNPs (Uitdewilligen et al. 2013)	37	32	28	87.5
SolCAP 8303 array (Felcher et al. 2012)	4179	3788	3561	94.0
SolCAP 69K detection (Hamilton et al. 2011)	284	246	202	82.1
Candidate genes	124	110	32	29.1
Total	19,747	17,978	14,530	80.8

### Plant materials

In this study we report about 639 DNA samples hybridized on the SolSTW array, of which 569 were unique genotypes consisting of 537 tetraploids and 32 diploids. To analyse the reproducibility, 39 tetraploid and 5 diploid samples were replicated on the array using DNA isolated from plants obtained from different sources. Besides these replicates the DNA from a single DNA isolation of clone RH89-039-16 (26 times) was used as an internal standard. The tetraploid genotypes consisted of 192 genotypes of a representative subset of commercial potato germplasm available worldwide, selected for the study of (D’hoop et al. 2008) and complemented with a set of 173 advanced breeding lines from Dutch potato breeders described in D’hoop et al. 2011, 2014, respectively. An additional set of 171 genotypes was collected, consisting of 51 cultivars and 120 advanced breeding lines provided by Dr. Ronald Hutten (Wageningen UR Plant Breeding) and the company Meijer B.V. The names and additional information of the genotypes are provided in supplementary file 2.

### Data collection: DNA

Leaf material was collected for DNA extraction using the Thermo Scientific KingFisher Flex. DNA concentration was measured with the NanoDrop spectrophotometer and the DNA concentration was adjusted to ~50 ng µL^−1^ when possible. When DNA concentration was lower than 50 ng µL^−1^ the sample was still used up to a minimum of 25 ng µL^−1^ whilst for samples with lower concentrations a new DNA isolation was performed. For each 96-well plate the diploid genotype RH89-039-16 was included as a control. Infinium arrays were processed according to the manufacturer’s suggested protocol at ServiceXS, Leiden, the Netherlands.

### Genotype calling with fitTetra

For the genotype calling, fitTetra (Voorrips et al. 2011) and Illumina GenomeStudio software (version 2010.3, Illumina, San Diego, CA) were used. Whilst the polyploid module of GenomeStudio requires manual determination of the position and boundaries of the five clusters for each marker separately, fitTetra can perform this task fully automated. Therefore fitTetra was used to automatically score all markers. GenomeStudio was only used when the clustering of fitTetra resulted in inadequate genotype calling according to the criteria described below.

fitTetra first removes all data points with an overall R-value (overall intensity) below 0.2. Subsequently two default settings of fitTetra were adjusted (1) p.threshold was lowered from 0.99 to 0.95, which implies that there is 95 % confidence of a sample belonging to a cluster, resulting in less missing calls as compared to the more strict 0.99 threshold. (2) The peak.threshold was increased from 0.85 to 0.99, which allows SNPs with a very low allele frequency (up to 99 % of all markers in 1 genotypic class) to be fitted by fitTetra. This was needed because the design of the array included a high number of low frequent SNPs. (3) the call.threshold was set to 0.60, resulting in the rejection of markers with more than 40 % missing values. Diploid samples are analysed along with the tetraploids to allow verification of the correct recognition of the nulliplex (AAAA), duplex (AABB) or quadruplex (BBBB) clusters.

**Fig. 2:**
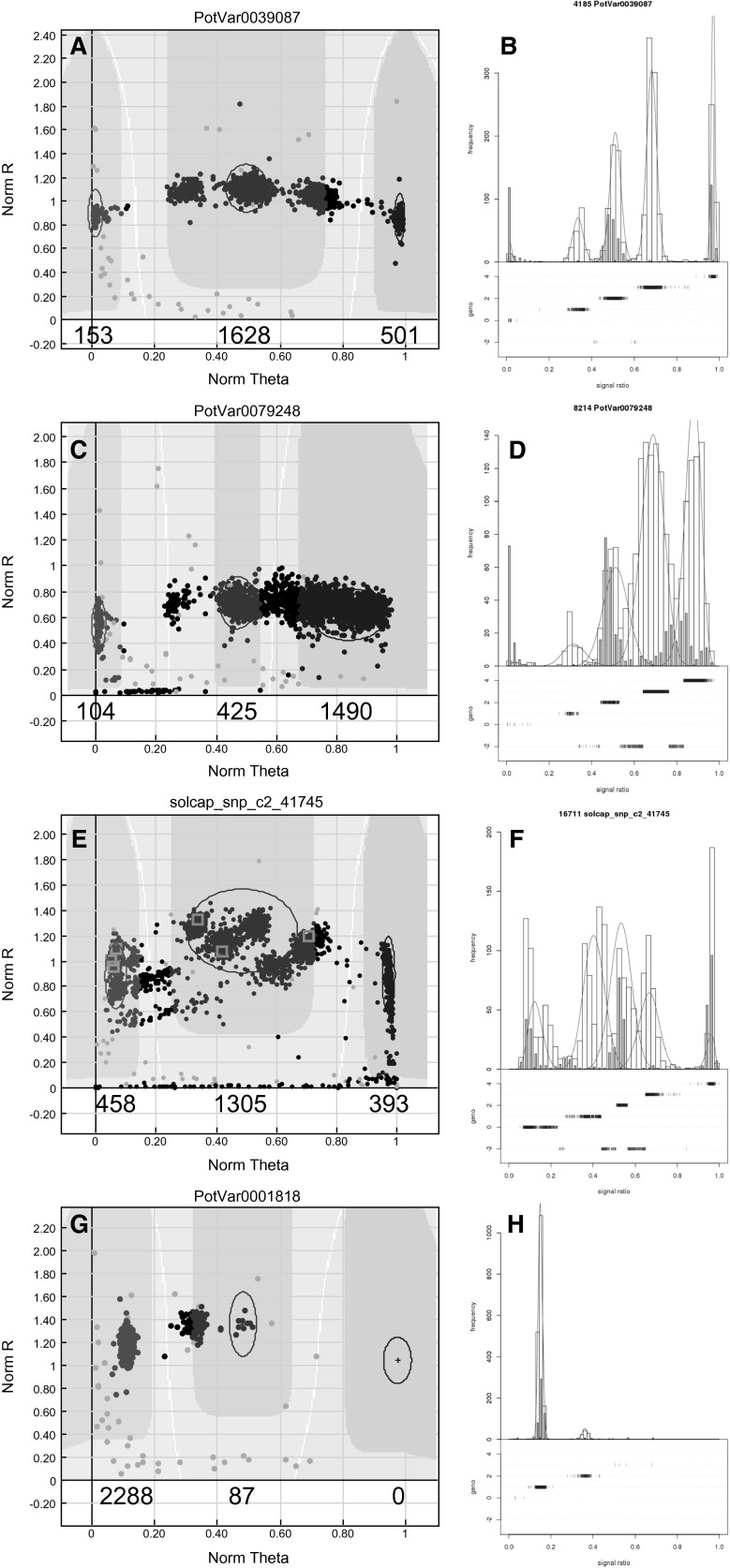
GenomeStudio (*left*) and fitTetra (*right*) output. In panel **a** and **b** an ideal marker is visible, where five clusters are clearly distinguishable and the diploid samples (*grey bars*) cluster in the nulliplex, duplex and quadruplex clusters. In panel **c** and **d** a marker with a “cloud” of data points with overlap between clusters is visible, in panel **e** and **f** a marker with >5 clusters is visible and in panel **g** and **h** a marker with the nulliplex cluster shifted to the *right* is visible. *Grey bars* in fitTetra output represent diploid samples, these should cluster in three groups as in **a**. *Blue bars* in the bottom of fitTetra output represent the dosage calls and *red bars* represent genotypes which are in-between clusters (color figure online)

Simultaneous analysis of diploids and tetraploids may however compromise Hardy–Weinberg assumptions implemented in fitTetra and this may result in the rejection of markers that display deviations from Hardy–Weinberg equilibrium. Therefore two runs were performed, one with and one without the diploid samples. Genotype calls of both fitTetra runs were compared and inspected for markers having obvious genotyping errors such as (1) a heterozygous genotype call for the reference genome genotype DM; (2) diploid genotype calls assigned to simplex or triplex clusters and (3) deviating Mendelian segregation in a tetraploid mapping population from a matching project (analysis of the tetraploid mapping population is beyond the scope of this paper). Markers showing one of these unexpected results could be the result of a poor marker or a poor clustering by fitTetra. The poorly clustered markers along with the chloroplast markers were manually scored with GenomeStudio. Additionally SNP markers initially rejected by fitTetra were visually inspected using the graphical output of fitTetra (Fig. 2) to diagnose the correctness of the rejection. SNP markers, rejected by fitTetra, but allowed manual scoring were re-joined with the final dataset using GenomeStudio.

Genotype calling with fitTetra results in dosage scores (0, 1, 2, 3 or 4) which reflects the Infinium assay design by the manufacturer, which uses the A nucleotide as a reference. Genotype calls were converted from the initial Illumina format into two derived datasets. The first dataset (used for association analysis) is based on the DM reference genome, where the SNP alleles are indicated as REF (DM) and ALT (non-DM). SNP dosage values ranging from 0 to 4 reflects the observed number of non-DM SNP alleles. The second dataset (used for population genetic analysis; this study) is based on the population minor allele frequency (MAF), where the SNP alleles are indicated as MIN (minor allele) or MAJ (major allele). SNP dosage values in this second dataset ranging from 0 to 4 reflect the observed dosage of the minor allele. This MAF dataset is more convenient, because none of the many haplotypes in the potato gene pool assumes a haplotype frequency exceeding 50 % (Uitdewilligen et al. 2013).

### SNP dating to identify introgression breeding

In the second SNP dataset (MAF) the SNP dosage specifies the presence of specific SNP alleles. Subsequently we searched for the oldest cultivar that had this minor SNP allele. The age of a genotype is based on the year of market release as listed in the potato pedigree database (Van Berloo et al. 2007). For progenitors/unnamed genotypes the year of crossing plus 10 years was taken. In this way the genotype and the year of introduction of each SNP was estimated. SNPs polymorphic in one of the 48 cultivars released before 1945 are defined as “pre-1945” genetic variants. These SNP markers usually continue to be polymorphic after 1945. SNPs being monomorphic before 1945 and polymorphic in one or more of the 489 genotypes released after 1945 are assumed to be the result of introgression breeding and are defined as “new” or “post-1945” variants. The year 1945 was chosen because in 1946 Craigs Bounty was released, which is the oldest cultivar in our dataset harbouring wild species in its pedigree.

### Analysis of changes in genetic composition of the potato gene pool

Changes in allele frequency were calculated to study the effect of breeding on the genetic composition of the gene pool. For this purpose the allele frequencies in the group of cultivars released before 1945 (*N* = 48) was compared with the allele frequency in cultivars released since 2005 (*N* = 81) and the allele frequency in the “starch” sub-populations (*N* = 59). Additionally the effect of allele dosage in important founders was compared with allele frequency changes. The subsets of genotypes are specified in supplementary file 2.

## Results

### Development of the SolSTW SNP array

The SolSTW SNP array combines SNPs from two discovery studies (Hamilton et al. 2011; Uitdewilligen et al. 2013). SNPs originating from Hamilton et al. (2011) were selected based on good performance in an earlier experiment using the SolCAP SNP array (data not shown) without any additional selection criteria. In contrast, the large set of 129,156 SNPs originating from Uitdewilligen et al. (2013) required stringent selection criteria since only a small subset could be selected. The high SNP density in potato allowed us to narrow down the number of potential SNP assays to 59,279 SNPs. Subsequently, redundancy amongst SNPs was reduced by clustering all SNPs according to SNP dosage as described by Uitdewilligen et al. (2013). This resulted in 7019 clusters and 5334 single SNPs (singletons). For around 5200 clusters, two or more SNP per cluster and gene were selected. Of the remaining approximately 1800 clusters, one SNP was selected and complemented with 2738 singletons, resulting in a total of 15,123 selected SNPs. SNPs originating from Uitdewilligen et al. (2013) will be referred to as PotVar SNPs. In Table 1 the attempted numbers of SNPs are shown.

### Optimization of fitTetra with SolSTW array

Several runs were performed with fitTetra for genotype calling using the signal ratios obtained from the Infinium array. Over sequential runs, the programme settings were optimized and minor errors of the software were corrected. Two properties of the Infinium data initially resulted in erroneous clustering by the software. Firstly it appeared that the five clusters are not evenly distributed over the X-axis, as shown in Fig. 2a, b. In particular the three heterozygous clusters are closer to each other and relatively far from the two homozygous clusters. Secondly, the signal of the homozygous clusters is biased and not exactly at 0 or at 1 as shown in Fig. 2g, h. These modifications of the software are processed in the publically available version of fitTetra since autumn 2013 (https://www.wageningenur.nl/en/show/Software-fitTetra.htm).

### Analysis of the SolSTW array with optimized fitTetra software

The improved version of fitTetra was used for the genotype calling of the SolSTW array. The genotype calling was performed twice, once using all genotypes and a second run without the diploid genotypes. The genotype calling without the diploid samples was used for further analysis, as inclusion of the diploid samples resulted in an additional rejection of 1184 markers, due to deviation from a Hardy–Weinberg test by fitTetra. The analysis of the tetraploid samples resulted in 15,271 fitted and 2716 rejected markers. Subsequently, a bi-parental tetraploid mapping population was used to identify SNPs where parental SNP dosage and offspring ratios were in disagreement. This is a putative indicator of poor SNP performance, and visual inspection of GenomeStudio output as shown in Fig. 2 resulted in the rejection of another 378 SNPs. In addition 1832 markers with a call rate below 95 % in fitTetra were visually inspected using GenomeStudio. The remainder of 6041 SNPs with good Mendelian fit and call rate >95 % were assumed to be good calls, and visual inspection was omitted. For the visual inspection fitTetra output was used as shown in Fig. 2b, d, f, h. In these figures diploid samples are illustrated with grey bars. The position on the X-axis of the diploids allows one to identify potentially poor markers, when diploid samples are in simplex or triplex clusters. As shown in Fig. 2d, f the diploid samples do not cluster together in the nulliplex, duplex or quadruplex clusters and therefore markers like these were removed. This incorrect clustering of diploids was predominantly observed in markers with more than 5 clusters as shown in Fig. 2e, f or markers with “clouds” of data points as shown in Fig. 2c, d. For 1206 of the 1832 markers with >5 % missing calls, visual inspection resulted in the removal of the markers from the final dataset. For 626 markers, fitTetra produced false negative genotype calls based on correct marker signal intensities. Such markers were manually re-scored using GenomeStudio. The 2716 rejected markers were visually inspected with fitTetra output as shown in Fig. 2, and scored manually if the marker was mistakenly rejected. This resulted in the recovery of 843 markers. Of these 843 markers 689 had an allele frequency below 1 %, therefore these were correctly rejected based on the peak.threshold setting in fitTetra of 0.99. The remaining 154 were mistakenly rejected for unknown reasons.

### Reproducibility of genotype calls

As shown in Tables 1 and 2 the data collection with fitTetra and GenomeStudio resulted in a final dataset with a high number of 14,530 SNP markers. The genotype calls of the 39 replicated tetraploid samples showed a high concordance between replications. On average, only 3.3 calls (0.02 %) differed between the replicated samples of which 60 % are differences within the heterozygous clusters. Additionally for 74 (0.5 %) markers on average there was no call for either of the genotypes. The 26 replicates of the internal diploid control also showed highly concordant results. We observed seven markers with a deviating observation. In addition, we observed 66 markers with one or more missing calls, of which 50 % were caused by two of the twenty-eight replicates.

**Table 2:** Summary of total number of SNPs

Chromosome	PotVar		SolCAP		Total	% New
	Pre-1945	Post-1945	Pre-1945	Post-1945		
St4.03ch00^a^	42	7	45	1	95	131
St4.03ch01	902	478	405	17	1802	27.5
St4.03ch02	840	405	316	50	1611	28.1
St4.03ch03	662	307	293	45	1307	26.9
St4.03ch04	767	305	382	8	1462	21.4
St4.03ch05	830	254	214	24	1322	21.0
St4.03ch06	524	177	277	6	984	18.6
St4.03ch07	524	318	333	20	1195	282
St4.03ch08	485	154	334	4	977	16.2
St4.03ch09	535	206	302	9	1052	20.1
St4.03ch10	385	179	206	1	771	23.4
St4.03ch11	498	291	247	12	1048	28.8
St4.03ch12	479	185	190	22	876	23.6
Chloroplast	13	15	–	–	28	53.6
Total	7486	3281	3544	219	14,530	24.1

Numbers of SNP markers per chromosome separated per origin (PotVar, SolCAP) and SNP age (pre-, or post-1945). Manually developed markers are within the set of PotVar markers

^a^St4.03ch00 lists marker that are located on unanchored scaffolds of the reference genome

The percentage of missing calls was very low for the final dataset of 14,530 markers and 537 genotypes, with only an average of 95 missing calls per genotype and 3.5 missing calls per marker (0.65 %). For genotypes having wild species in their pedigree and not used in the SNP discovery panel of Uitdewilligen et al. (2013), the average number of missing values was much higher (184).

### Analysis of factors influencing assay failure

Several possible factors that could cause assay failure have been examined. In Table 1 percentages of assay failure are shown based on the origin of the SNP assay. What is clearly visible is that the SolCAP SNPs originating from the 8303 array are most successful (94.0 %), because these SNPs were tested before with the Infinium platform. The non-pre-tested SNPs from Hamilton et al. and the SNPs originating from the SNP discovery study of Uitdewilligen et al. (2013) show a lower percentage of successful assays (82.5 and 77.5 %, respectively). However, when considering markers in coding regions only, the assay failure rate of PotVar SNPs is much lower (11.4 %, Table 3). For SNPs that were manually developed the majority failed (70 %), this could be explained by the location in R-genes, which are members of a large highly variable gene family. In Table 3 percentages of assay failure of 12,272 SNPs are shown based on their localization in coding or non-coding regions, as well as based on their chromosomal position on the pseudomolecules (Sharma et al. 2013). The latter can be divided in euchromatin, pericentromeric heterochromatin and the border between the two. It is clear that SNPs localized in the pericentromeric heterochromatin are more likely to fail. However, more significant is the low percentage of assay failure in coding regions compared to non-coding regions.

**Table 3:** Assay failure as a function of chromosomal positions for PotVar SNPs

Position on pseudomolecule	Coding/non-coding	Ok	Failed	Percentage
Euchromatin	Coding	4348	538	11.0
Border	Coding	313	46	12.8
Heterochromatin	Coding	221	42	16.0
Total (coding)		4882	626	11.4
Euchromatin	Non-coding	3828	1214	24.1
Border	Non-coding	318	216	40.4
Heterochromatin	Non-coding	449	739	62.2
Total (coding + non-coding)		9477	2795	22.8

Number and percentage of successful and failed SNPs separated based on position on the pseudomolecules (Euchromatin, heterochromatin and border as defined by Sharma et al. 2013) and based on coding and non-coding regions

**Fig. 3:**
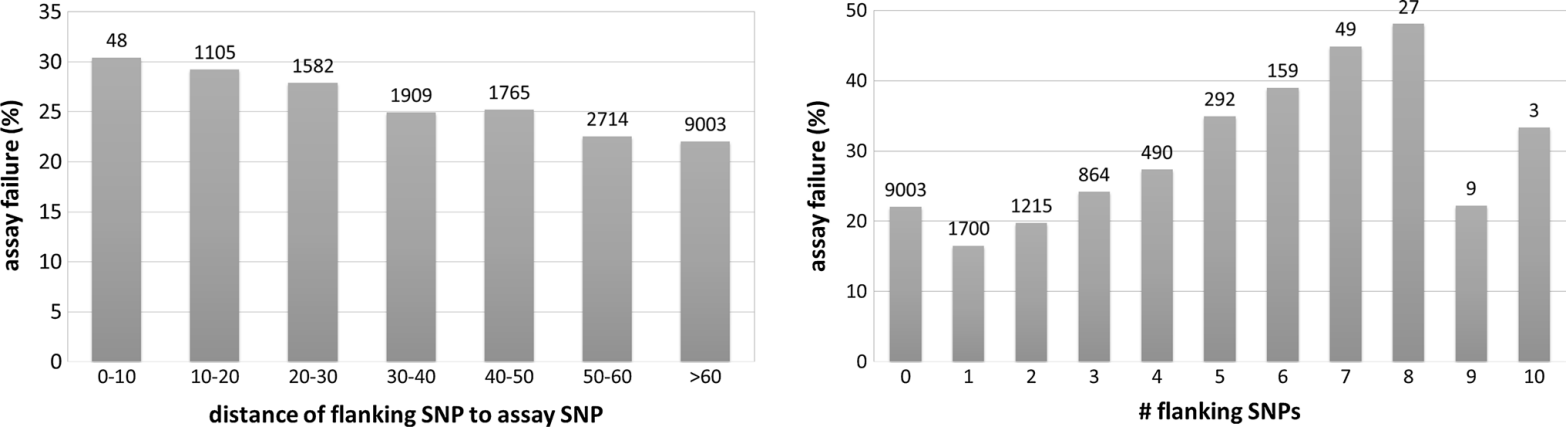
Assay failure as function of flanking SNPs. (*Left panel*) Percentages of assay failure as function of the distance (in bp) of the first flanking SNP to the attempted SNP assay. (*Right Panel*) Percentage of assay failure as a function of the total number of SNPs observed in 50 bp flanking region

The high nucleotide diversity of potato implies that SNP assays may be frequently affected by flanking SNPs. Therefore we aimed to target SNPs without flanking SNPs for assays, this is however problematic in potato due to its high SNP density. Consequently for many (34.8 %) SNP assays (originating from Uitdewilligen et al. 2013) on this array, known flanking SNPs are present. In Fig. 3a the percentage of assay failure of these PotVar SNPs is shown as a function of the distance of the flanking SNPs. This graph shows a trend where flanking SNP distance is correlated with assay failure. Additionally in Fig. 3b a correlation is shown between assay failure and the number of flanking SNPs. An increase in assay failure with more flanking SNPs can be observed. In addition the GC content was compared between successful and failed SNPs, however there was no significant relation between assay failure and GC content.

### Allele frequencies

**Fig. 4:**
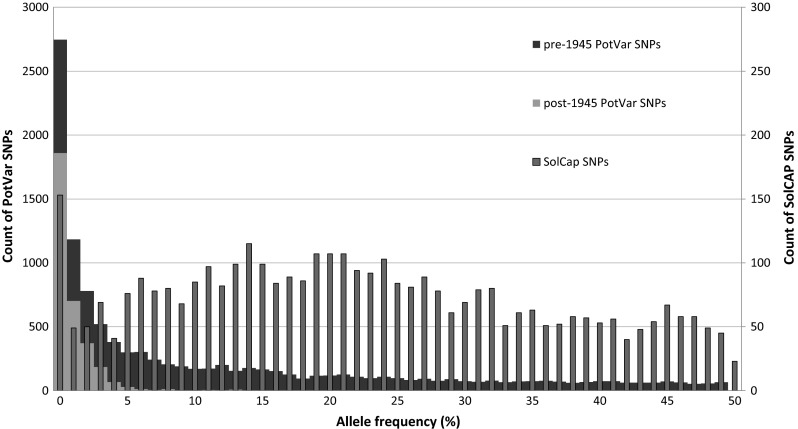
Allele frequency distribution. Frequency distribution of Minor Allele Frequencies of SNPs in 537 tetraploid genotypes. The *wide bars* display the distribution of the 10,707 PotVar markers (Uitdewilligen et al. 2013), where the *blue* part indicates the proportion of pre-1945 SNPs and *green* the post-1945 SNPs. The distribution with *narrow* bars displays the Minor Allele Frequencies of the 3574 + 188 SolCAP markers (Hamilton et al. 2011). Chloroplast and manually developed markers are not included in this figure. The *left Y-axis* is for the PotVar SNPs and the *right Y-axis* is for the SolCAP SNPs (color figure online)

The allele frequency distribution of SNPs across the 537 genotypes is shown in Fig. 4. PotVar SNPs, shown in the distribution (wide bars, left Y-axis) and SolCAP SNPs (narrow bars, right x-axis) differ greatly in allele frequency. PotVar SNPs are split in pre-1945 (dark blue) and post-1945 (green) SNPs. The average allele frequency of PotVar SNPs is 11 % and for SolCAP 22.7 %. This large difference in allele frequencies, also shown in Table 4, is not surprising since we deliberately did not exclude SNPs with a low allele frequency, clearly these were selected against in the design of the SolCAP array.

**Table 4:** Numbers and average minor allele frequencies (MAF) of SNPs by age (polymorphic in pre-, post-1945 cultivars) and discovery study [PotVar from Uitdewilligen et al. (2013), SolCAP from Hamilton et al. (2011)]

		Pre-1945 SNPs	Post-1945 SNPs	Total
PotVar SNPs	Numbers Average MAF	7486 15.2 %	3281 1.3 %	10,769 11.0 %
SolCAP SNPs	Numbers Average MAF	3544 24.1 %	219 1.5 %	3763 22.8 %
Total	Numbers Average MAF	11,030 18.0 %	3500 1.4 %	14,530 14.0 %

### Identification of pre-1945 and post-1945 variation

**Fig. 5:**
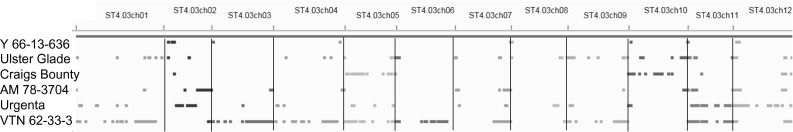
Genomic position of newly introduced variation. Genome-wide plot of the coordinates of post-1945 SNPs on the DM reference genome where post-1945 SNP indicates the position of putative introgression segments first observed in six cultivars.* Each* *dot* represents one SNP and it is visible that multiple cultivars can introduce different haplotypes in the same region

The comprehensive sampling of the gene pool of cultivated potato allowed the evaluation of changes of the composition of the gene pool over time. This resulted in the identification of SNP markers, which are the result of introgression breeding and SNP markers that represent the initial genetic diversity within the founders of the contemporary gene pool. A SNP that is polymorphic in one of the 48 cultivars released before 1945 is hereafter referred to as “pre-1945” SNP. This genetic variation most likely represents the material that was brought to Europe from the Americas between the 16th and the 19th century. A SNP marker that is monomorphic in one of the 48 old cultivars, but polymorphic in more recent cultivars/progenitors is hereafter referred to as “post-1945” variation. In Table 4 the large difference in allele frequency is visible between the post-1945 SNPs (average MAF = 1.4 %) and the pre-1945 SNPs (average MAF = 18.0 %). In Table 2 the numbers and percentages of post-1945 SNPs per chromosome are shown. In total 3500 (3281 PotVar + 219 SolCAP) SNPs are post-1945, which corresponds to 24.1 % of the SNP markers in this array. The detection study of Uitdewilligen et al. (2013) made a large contribution to this group of post-1945 SNPs (Table 2). The 219 post-1945 SNPs contributed by SolCAP are mostly introduced by cultivar Lenape (114 SNPs), of which two descendants (Atlantic and Snowden) were included in the discovery study of Hamilton et al. (2011). The chromosomal positions of post-1945 SNPs were analysed. It appears that post-1945 SNPs cluster together on chromosomes and in genotypes. In Fig. 5, a genome-wide plot is shown of the location of introgression segments first observed in six genotypes. Introgression segments differ greatly in size, ranging from very small (Y-66-13-636) to complete chromosomes (VTN 62-33-3). A nice example is the 97 SNPs first observed in Craigs Bounty (1946). This figure shows 95 SNPs in three introgression segments on chromosomes 5 (green), 10 (dark blue) and 12 (grey). Ten genotypes (VTN 62-33-3, Lenape, Mara, Urgenta, VE 71-105, AM 78-3704, Maris Piper, Craigs Bounty, Ulster Glade, VE 66-295) are responsible for the introduction of 50 % of post-1945 SNPs. A full table with numbers of SNP introduced per cultivar is shown in supplementary file 3.

### Processes that shape the genetic composition of the contemporary gene pool of potato

**Fig. 6:**
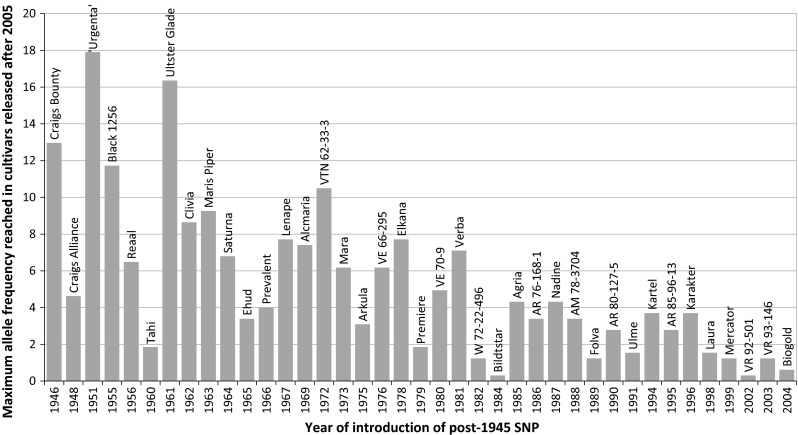
Positive selection on newly introgressed SNPs. This figure shows the maximum allele frequency of post-1945 SNP reached in a panel of 244 genotypes released since 2005. The higher the *bar* the more frequent the SNP is observed in recent material, suggesting positive selection. The cultivar name above* each* *bar* indicates the first cultivar on the market showing a polymorphism and thus represents the founder genotype of the SNP

Several processes are shaping the contemporary gene pool of potato, such as the introduction of new genetic variants by introgression breeding. Introgressions cause the loss of existing variants by substitution. Selection will also influence the allele frequency, including breeding for specific market niches (e.g. starch cultivars). In specific market niches, the limited gene pool is easily affected by random genetic drift (genetic erosion). These processes (introgression/substitution, selection, drift) were studied by comparing SNP allele frequencies between two groups. Firstly, the pre-1945 cultivars were compared with the cultivars released after 2005. Also, the pre-1945 cultivars were compared against cultivars from the “starch” subpopulation. For post-1945 SNPs significant increases of the allele frequency can be observed. In this study we analysed 246 cultivars that were released between 1946 and 2005. In this group, 108 cultivars contributed post-1945 SNPs, ranging from 1 to 447 post-1945 SNPs per cultivar (Supplementary file 2). From these 108 cultivars 39 are shown in Fig. 6 and arranged in the order of market introduction. These 39 cultivars are donors of those post-1945 SNPs that have attained the largest increase in allele frequency within the 242 cultivars released after 2005. The negative slope perceived in Fig. 6 indicates that introgression segments introduced soon after 1945 could assume a higher allele frequency (up to 19 %) as compared to more recently introgressed haplotypes (up to 4 % increase). This suggests that a prolonged presence of a beneficial haplotype introgressed in the gene pool results in increasingly higher allele frequencies due to positive selection. Please note that a 4 % increase in allele frequency implies that almost 20 % of the cultivars carry this haplotype in simplex condition, whereas a 19 % increase implies that more than half of the cultivars are simplex or duplex and occasionally triplex.

In contrast, 50 % of all post-1945 SNPs remain below an allele frequency of 1 % and 549 SNPs were not polymorphic anymore (nulliplex) in cultivars released after 2005. These 549 SNPs could be considered as lost, i.e. phased out soon after introduction. For the pre-1945 SNPs, 538 SNPs (4.9 %) were no longer polymorphic in contemporary cultivars. These SNPs are also assumed to be lost during breeding. This may be due to selection, but random genetic drift is also plausible, because the initial allele frequency of these SNPs in old germplasm was already very low (1.4 % on average).

**Fig. 7:**
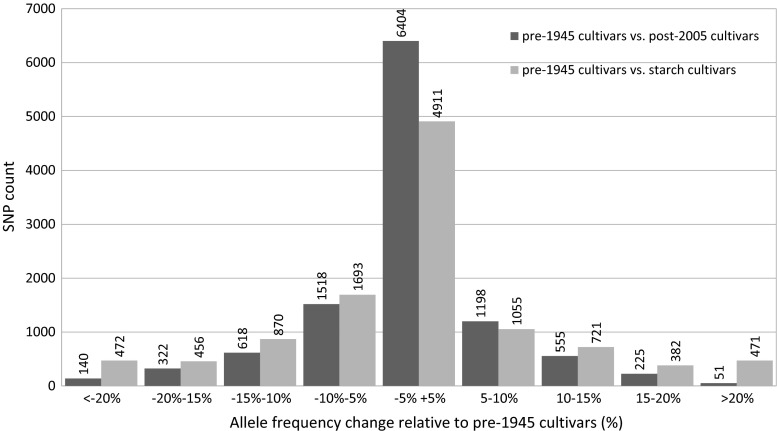
Allele frequency changes. Distribution of allele frequency change of all pre-1945 SNPs is shown. The *dark grey bars* represent the number of SNPs and their change in minor allele frequency as compared between a panel of older cultivars (market release before 1945) and a panel of new cultivars (market release after 2005). The *light grey bars* show the comparison between older cultivars and genotypes included in the subpopulation of starch cultivars

**Fig. 8:**
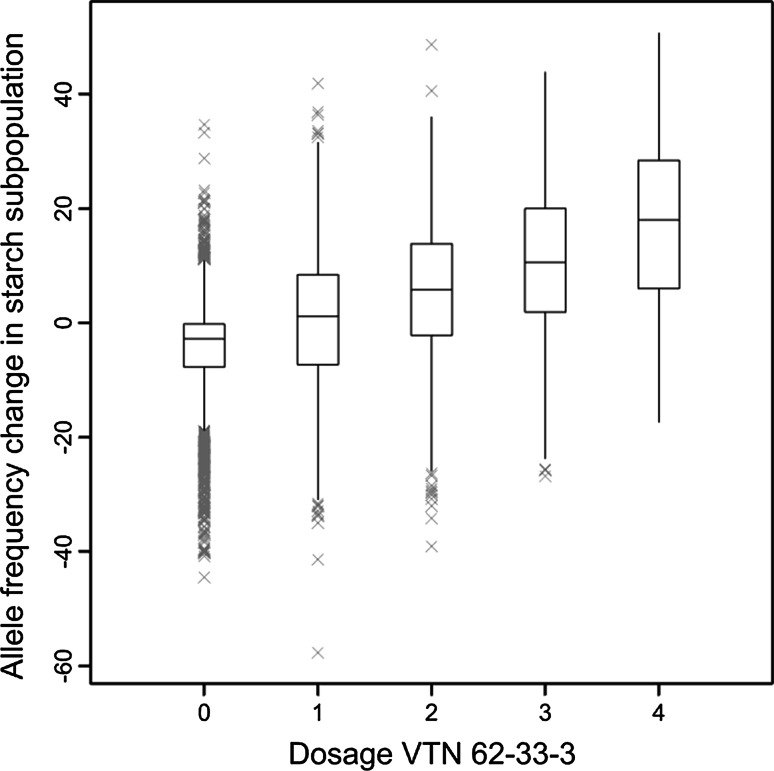
Founder effect. The change of the Minor Allele Frequencies of pre-1945 SNPs within the subpopulation of starch cultivars compared with pre-1945 cultivars (*Y-axis*) as a function of the allele dosage of the minor allele in one of the most important progenitor clones (VTN 62-33-3). Here evidence is provided that a founder effect (initial allele dosage of a pre-1945 SNP) has a substantial impact, comparable to selection of a post-1945 SNP

A comprehensive overview of the changes in allele frequency of all pre-1945 SNPs (in post-2005 and starch cultivars compared with old cultivars) is shown in Fig. 7. The largest column in the middle of the figure shows that the majority of the SNPs (6441 or 42 %) hardly changed in allele frequency during a century of potato breeding. Starch cultivars show somewhat larger fluctuations in allele frequencies, because of an emphasis on introgression breeding for nematode resistance along with founder effects (discussed below). Figure 7 also suggests that larger numbers of SNPs have declined, as compared to the number of SNPs that show an increased allele frequency. This suggests that broadening of the genetic diversity by introgression since 1945 results in an overall net decrease of the frequency of pre-1945 haplotypes. In addition to these allele substitutions, founder effects may also reinforce this fluctuation. In Fig. 8 the change in allele frequency in the “starch” subpopulation is plotted against the allele dosage of an important progenitor (VTN 62-33-3). The figure clearly shows that a higher dosage of a SNP in an important founder contributes to the gain in allele frequency over time. The correlation between SNP dosage in a specific founder and allele frequency gain within the “starch” subpopulation was strongest for VTN 62-33-3 and AM 78-7804, two frequently used progenitors.

The processes underlying allele frequency changes over time: introgression, substitution, selection, drift and founder effects (frequent use of parents) are highly confounded. Still we assume that SNP variants that show the greatest increase in frequency are linked to important alleles for agronomic performance and vice versa. The most striking observations are that (1) 95.1 % of the pre-1945 SNPs are still polymorphic after 50–150 years of breeding and (2) we do not observe any fixation of pre-1945 SNPs in cultivars released after 2005.

## Discussion

### Ascertainment bias

Here, we describe the development and evaluation of a SNP array for potato. Another SNP array named SolCAP is already widely used by the scientific community (Felcher et al. 2012; Hirsch et al. 2013; Lindqvist-Kreuze et al. 2014; Manrique-Carpintero et al. 2014; Prashar et al. 2014). We acknowledge the value of this array and reused 4179 SNPs with good assay performance. The other SNPs of this 20K SolSTW array were from a discovery panel of 83 tetraploids, comprising progenitors and cultivars across breeding history, geography and market niche (Uitdewilligen et al. 2013).

Genetic diversity is unequally distributed across the gene pool. Therefore, a systematic deviation of a SNP discovery panel relative to the set of interrogated individuals will result in an ascertainment bias. Where SolCAP retrieved SNPs predominantly from North American cultivars for the processing industry, the discovery panel of Uitdewilligen et al. (2013) contributed a relatively high number of SNPs typical for wild species introgression segments in progenitors. Although ascertainment bias is an important issue in the development and application of SNP arrays (Moragues et al. 2010; Thomson et al. 2012), it is difficult to quantify and difficult to avoid. A wider discovery study is in general better for SNP arrays intended for a wide range of applications. In view of the progenitor clones included in the discovery panel this array will be valuable to identify SNPs associated with resistance to cyst nematodes and viruses, but the array will be blind for East European haplotypes conferring e.g. resistance to Wart pathotype 18.

### Assay quality

In view of the high SNP density in potato (Uitdewilligen et al. 2013) a comprehensive SNP discovery panel also allows the identification of flanking SNPs that could negatively influence SNP assay quality. Unavoidably, 34.8 % of the markers have a known SNP flanking the SNP assay and Figs. 2 and 3 illustrate their impact on assay quality. The analyses confirmed the negative effect of flanking SNPs, but also that their effect is proportional to the proximity and the amounts of flanking SNPs. Surprisingly, genomic position was a much stronger indicator for assay failure, where SNPs located outside exons or beyond gene-rich euchromatic regions were more likely to fail.

### Data collection with fitTetra

In contrast to automated genotype calling in diploids, genotype calling in tetraploids is not a trivial job. Standard software such as GenomeStudio can handle tetraploid data, but it cannot automatically cluster the fluorescent signal into the five potential clusters. Manual scoring of each marker separately will give the best results, however due to the increasing size of SNP arrays the workload of manual scoring is prohibitively high.

Several methods have been proposed to automatically score tetraploid data. Hackett et al. (2013) used an algorithm to cluster ratios based on the genotype call of the parents in a segregating population. In the paper of Hirsch et al. (2013) a custom cluster file was generated within GenomeStudio. Here, prior knowledge of the clusters per marker is needed, since every marker produces a slightly different distribution of the five clusters. Since this was the first time the array was analysed, such a custom cluster file is not available. Consequently, we used software specifically designed for genotype calling of tetraploids, fitTetra (Voorrips et al. 2011) and were able to gain more experience with this software. During the analysis of our data a number of improvements in the fitTetra software could be implemented, as described in the results. We recommended users to always download the latest software version from our website (https://www.wageningenur.nl/en/show/Software-fitTetra.htm). With fitTetra we were able to score the dosage for 80.5 % of the markers. This is relatively high compared to 45 % in Hirsch et al. (2013) and 38 % in Lindqvist-Kreuze et al. (2014). Furthermore, the clustering by fitTetra appeared to be more accurate than genotype calling by GenomeStudio. The 0.02 % difference between replicated samples reported in our study compares well with the 1.7 % difference in genotype calls reported before (Hirsch et al. 2013). Nevertheless, we show that fitTetra does not always assign the correct genotype call to a cluster (Fig. 2g, h), or will erroneously cluster poor markers (Fig. 2c, f). Inclusion of diploid samples and a tetraploid bi-parental mapping population were extremely helpful to identify and discard poor SNP assays. Without these internal controls the quality of our dataset is expected to be much lower. An additional advantage of fitTetra is that the visual output of fitTetra is very helpful for identifying poor assays.

### Dating of SNPs as a tool for reconstruction of the breeding history

A comprehensive sampling of genotypes of different ages has enabled us to assign a date to each SNP and to differentiate between “pre-1945” and “post-1945” genetic variants based on the year of market release. We observed that new genetic variants, cluster together in specific chromosomal regions and reside in specific genotypes (Fig. 5). For example, Craigs Bounty (released in 1946) is the oldest cultivar in our panel with introgressed chromosomal regions. In this cultivar, 97 SNPs are polymorphic, which were monomorphic in older pre-1945 cultivars. These post-1945 SNPs most likely descend from a (*S. commersonii* x S. *demissum*) × (S. *maglia* × *S. edinense*) hybrid six meiosis back in the pedigree (Van Berloo et al. 2007) and originate from the work of Salaman (1985). Craigs Bounty is one of the first cultivars with the *R1* gene conferring late blight resistance from *Solanum demissum* on chromosome *5* (Toxopeus 1956). Therefore the SNPs on chromosome *5* are good candidates for tagging the haplotype containing the *R1* gene. Subsequent linking of the pedigree to the first observation of CPC-1673 derived material (Maris Piper, 111 new SNPs) resulted in candidate SNPs on chromosome 5 tagging the *H1* resistance haplotype conferring resistance to *Globodera rostochiensis*.

The post-1945 SNPs, introduced with the market release of cultivar Lenape are most likely descending from the *Solanum chacoense* grand-grandparent. Why *S. chacoense* was used is not clear, but Love et al. (1998) describe Lenape as a first cultivar with a higher amount of solids, however it is also a cultivar with high glycoalkaloid content. Hence these SNPs could map nearby QTLs involved in dry matter content and/or glycoalkaloid content.

For most cultivars that contributed post-1945 SNPs the source of introgressions could be deduced from pedigree information. However, our data suggest that the cultivar Urgenta introduced 178 post-1945 SNPs. This does not match pedigree information describing Urgenta as a pure “*S. tuberosum*” cultivar. Along with the observation that Desiree, a daughter of Urgenta, does not contain any of the introgression segments, we conclude that this sample was named Urgenta erroneously.

### Processes that shape the genetic composition of the contemporary gene pool of potato

Few of the newly introgressed SNP alleles show a considerable increase in allele frequency within a subset of recent material (Fig. 6). Especially SNPs near the *H1* and *R3a/R3b* loci, reach an allele frequency of 15 and 10 %, respectively. This example of positive selection for SNPs flanking the *H1* locus can be explained by the need for cultivars resistant to *Globodera rostochiensis* by potato growers. The increase in frequency of SNP alleles that belong to the *R3a/R3b* haplotype is not easily understood. This locus *R3a/R3b* was soon overcome by late blight and does not provide a detectable level of field resistance to *Phytophthora infestans*. Nevertheless we observed that a large region (5 Mb) was retained in more recent material. This suggests that other beneficial alleles linked to these R-genes are introduced in the potato gene pool that caused a positive selection on alleles in this region, which might be interpreted as linkage drag. In contrast, the majority of the post-1945 SNPs do not exceed an allele frequency higher than 1 %. Since this variation is not under positive selection we conclude that this variation is not adding anything to the potato genepool and it will be a matter of time that this variation gets extinct in the newly introduced cultivars.

It is often thought that breeding will decrease the amount of genetic variation over time, also described as genetic erosion. However, this assumed trend of declining diversity is not supported by molecular data (van de Wouw et al. 2010). To our knowledge this is the first study that used dated SNPs to compare the loss of old polymorphisms with the influx of new diversity due to introgression. In agreement with van de Wouw et al. (2010), we observed an insignificant amount of genetic erosion in potato. The limited numbers of SNPs not being polymorphic anymore are most likely “lost” due to drift instead of selection against these SNP alleles. In fact the opposite is occurring. Whilst the majority of genetic variation that was present 100 years ago is still present in modern cultivars, new genetic variation introduced in the last decades caused an increase of genetic variation in the potato gene pool. The lack of fixation of beneficial alleles supports the hypothesis that breeders select highly heterozygous offspring, allowing optimal heterosis. The tetraploid nature of potato prevents efficient selection against non-beneficial alleles and the net result is that genetic erosion scarcely takes place. There are major shifts in allele frequencies also described by Hirsch et al. (2013), however only a limited set of SNPs show this pattern (Fig. 7). These more substantial changes in allele frequency can result from selection but can also be explained as a founder effect, where a higher allele dosage for SNPs along with the frequent use of an important progenitor has impact on the change of allele frequencies in breeding (Fig. 8). The joint effect of selection and founder effects may easily explain an allele frequency change up to 50 %.

### Future applications

This SNP array has been available for a short time, which is due to manufacturer’s quality criteria for shelf life, amount of material synthesized and willingness to keep stocks. We do not regret this short availability and will not re-order the same array. Arguments to avoid repetitive use of the same array are the ever-changing gene pool and the ever-changing ascertainment bias if the SNP discover panel is at odds with the QTL mapping panel. Finally, technology is evolving at high speed. Sequencing costs are dropping and bioinformatics tools become more user friendly to arrive at more cost-effective sequencing-based genotyping strategies. For future applications supplementary Table 1, attached to this publication, offers a lasting resource of SNP loci that have been demonstrated successful. As shown by Felcher et al. (2012) and here the initial success rate of a SNP assay ranges between 40 and 70 %. This publication confirms that a SNP assay, once sufficiently tested, has a very high probability of being good forever. Indeed, the inclusion of SNPs that were tested before with the SolCAP array were re-applied. For this group of SNPs a very high success rate was achieved of 94 %. Whenever there is a need to generate fixed SNP arrays or KASP assays, it is recommended to tap from SNPs that have been demonstrated as successful before.

## Author contribution statement

Conceived and designed the experiments: PGV, HJvE, JGAMLU. Performed the experiments: PGV. Analysed the data: REV, PGV. Wrote the manuscript: PGV, HJvE. Edited the manuscript: HJvE, REV, RGFV.

## Electronic supplementary material

Supplementary material 1 (XLSX 2336 kb)

Supplementary material 2 (XLSX 36 kb)

Supplementary material 3 (XLSX 12 kb)
